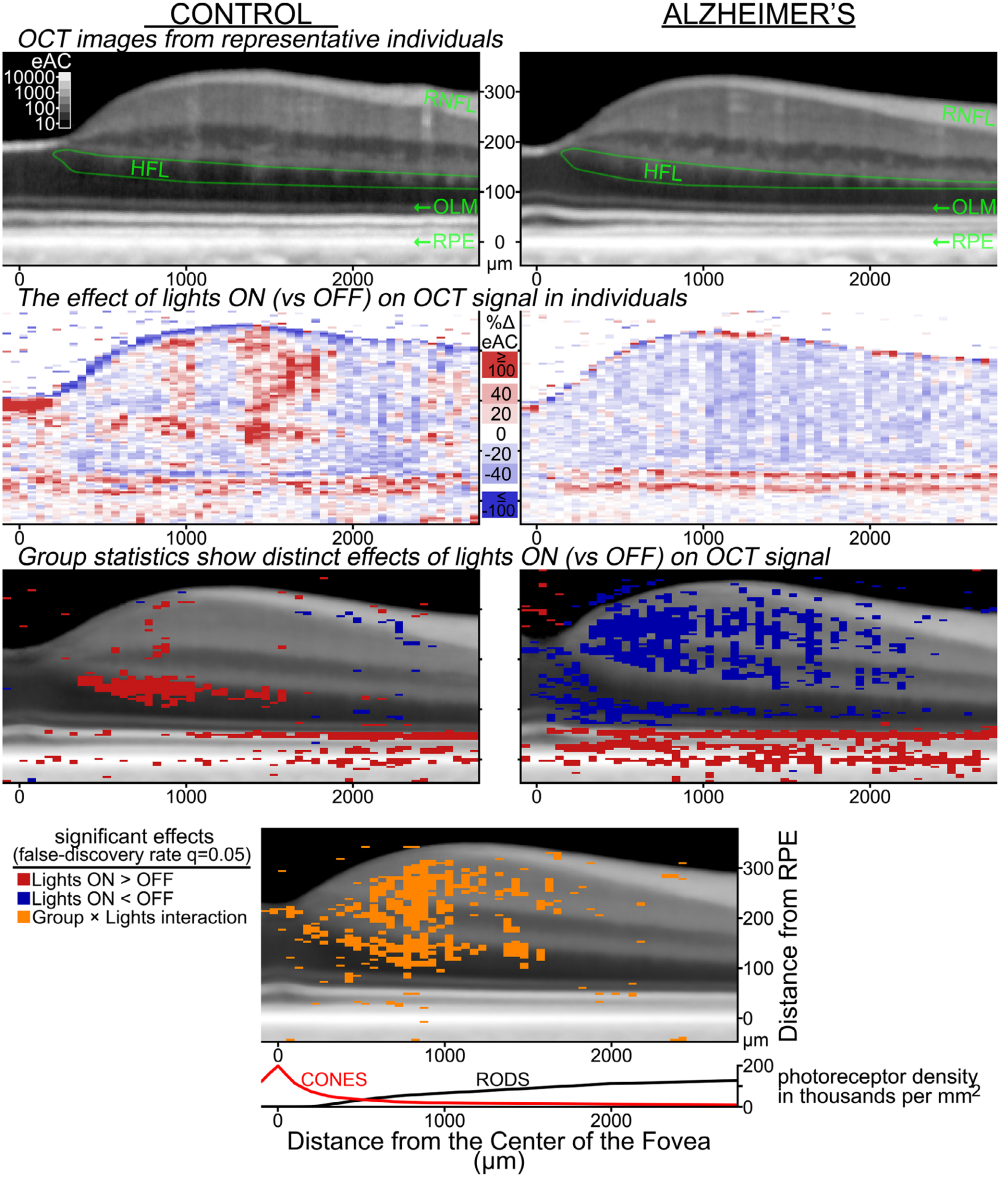# Abnormal light‐dependent retinal responses in early‐stage Alzheimer's disease

**DOI:** 10.1002/alz70856_101738

**Published:** 2025-12-24

**Authors:** Rita Mari Venua, David Philip Bissig

**Affiliations:** ^1^ University of California ‐ Davis, Sacramento, CA, USA

## Abstract

**Background:**

Glia facilitate amyloid beta clearance, which is reduced Alzheimer's disease (ALZ). In a prior optical coherence tomography (OCT) study of the retina, some light‐dependent changes co‐localized with macroglia, irrespective of neuronal and vascular/avascular boundaries. That putative marker of glial function was subtly abnormal in later stages of Alzheimer's disease (DOI: 10.1016/j.neuroimage.2020.117022). For this study, we (1) hypothesized that the same changes occurred at early stages, before retinal structural changes, (2) explored whether Alzheimer's‐associated functional abnormalities varied with proximity to the fovea.

**Method:**

We compared OCT images from 24 eyes of 13 people at an early stage of biomarker‐confirmed Alzheimer's disease (“ALZ”) to images from 19 eyes of 10 people without Alzheimer's (biomarker‐negative control; “CON”). After measuring macular thicknesses and peripapillary retinal nerve fiber layer thicknesses, we collected functional data – imaging in the darkness versus after bright light exposure. B‐scan intensities were transformed into attenuation coefficient maps, which we aligned and spatially normalized to facilitate groupwise comparisons. Multilevel models were used to summarize light‐dependent changes in each retinal layer spanning between the fovea and the optic disc.

**Result:**

ALZ and CON groups well‐balanced for age, sex, and education, but had different scores on the Montreal Cognitive Assessment (*p* <0.05; medians of 21 and 26, respectively). Only one structural difference was noted: the central 1 mm of macula was thicker in ALZ than in CON (296 vs 265 µm; *p* <0.0005). Both groups showed similar light responses over the rod and cone outer segments, but had significantly different (false discovery rate; q<0.05) functional responses in most other retinal layers; those occupied by macroglia. Group functional differences extended from cone‐only foveola through rod‐dominated perifovea, and were most robust 0.5‐1.0 mm from the foveal center.

**Conclusion:**

We find robust Alzheimer's‐related functional changes in the retina, but in an earlier disease stage than previously reported. The location of those changes implies a macroglial origin, rather than a primarily neural or primarily vascular origin.